# Identification and validation of a novel cellular senescence-related lncRNA prognostic signature for predicting immunotherapy response in stomach adenocarcinoma

**DOI:** 10.3389/fgene.2022.935056

**Published:** 2022-08-25

**Authors:** Cheng Zeng, Yu Liu, Rong He, Xiaohuan Lu, Yuyang Dai, Guoping Qi, Jingsong Liu, Jianzhong Deng, Wenbin Lu, Jianhua Jin, Qian Liu

**Affiliations:** ^1^ Department of Oncology, Wujin Hospital Affiliated with Jiangsu University, Changzhou, Jiangsu, China; ^2^ Department of Oncology, Wujin Clinical College of Xuzhou Medical University, Changzhou, Jiangsu, China; ^3^ Department of Internal Medicine, School of Medicine, Dalian Medical University, Dalian, Liaoning, China; ^4^ Cancer Institute, The Affiliated People’s Hospital of Jiangsu University, Zhenjiang, Jiangsu, China; ^5^ Department of Gastrointestinal Surgery, Union Hospital, Tongji Medical College, Huazhong University of Science and Technology, Wuhan, China

**Keywords:** cellular senescence, lncRNAs, immune infiltration, tumor mutation burden, microsatellite instability, stomach adenocarcinoma

## Abstract

**Background:** Cellular senescence is a novel hallmark of cancer associated with patient outcomes and tumor immunotherapy. However, the value of cellular senescence-related long non-coding RNAs (lncRNAs) in predicting prognosis and immunotherapy response for stomach adenocarcinoma (STAD) patients needs further investigation.

**Methods:** The transcriptome and corresponding clinical information of STAD and cellular senescence-related genes were, respectively, downloaded from the Cancer Genome Atlas (TCGA) and CellAge databases. Differential expression analysis and coexpression analysis were performed to obtain cellular senescence-related lncRNAs. Univariate regression analysis and least absolute shrinkage and selection operator (LASSO) Cox analysis were conducted to establish the cellular senescence-related lncRNA prognostic signature (CSLPS). Next, the survival curve, ROC curve, and nomogram were developed to assess the capacity of predictive models. Moreover, principal component analysis (PCA), gene set enrichment analysis (GSEA), tumor microenvironment (TME), tumor mutation burden (TMB), microsatellite instability (MSI), and tumor immune dysfunction and exclusion (TIDE) score analysis were performed between high- and low-risk groups.

**Results:** A novel CSLPS involving fifteen lncRNAs (REPIN1-AS1, AL355574.1, AC104695.3, AL033527.2, AC083902.1, TYMSOS, LINC00460, AC005165.1, AL136115.1, AC007405.2, AL391152.1, SCAT1, AC129507.1, AL121748.1, and ADAMTS9-AS1) was developed. According to the nomogram, the risk model based on the CSLPS was an independent prognostic factor and could predict 1-, 3-, and 5-year overall survival for STAD patients. GSEA suggested that the high-risk group was mainly associated with Toll-like receptor, JAK/STAT, NOD-like receptor, and chemokine signaling pathways. Further analysis revealed that STAD patients in the low-risk group with better clinical outcomes had a higher TMB, higher proportion of high microsatellite instability (MSI-H), better immune infiltration, and lower TIDE scores.

**Conclusion:** A fifteen-CSlncRNA prognostic signature could predict survival outcomes, and patients in the low-risk group may be more sensitive to immunotherapy.

## Introduction

Gastric cancer is the fifth most common malignant tumor globally, with over a million new cases in 2020 (Sung et al., 2021). Due to the insidious early symptoms of gastric cancer, most patients have entered the advanced stage at the time of diagnosis, making it the third leading cause of cancer-associated death (Smyth et al., 2020). Stomach adenocarcinoma (STAD) has a high mortality rate and is the most common histological type of gastric cancer. Precision medicine and immunotherapy have recently become hot spots in gastric cancer treatment. However, response rates of immune checkpoint inhibitors remain low (Yarchoan et al., 2017). Therefore, it is urgent to identify potential and beneficial individuals to increase the effect of immunotherapy on gastric cancer.

Cellular senescence, characterized by irreversible cell cycle arrest, is an essential aging phenotype and can accelerate organismal aging (Calcinotto et al., 2019). Studies have shown that cellular senescence is related to many diseases, including atherosclerosis, osteoporosis, glaucoma, neurodegeneration, and type 2 diabetes (Calcinotto et al., 2019). Recently, there has been growing interest in the role of cellular senescence in tumors. However, the role of cellular senescence in tumorigenesis and development remains controversial (Kowald et al., 2020). On the one hand, cellular senescence can activate innate and adaptive immune responses to limit tumorigenesis through the senescence-associated secretory phenotype (SASP), releasing large amounts of cytokines and chemokines (Reimann et al., 2010; Vicente et al., 2016), and on the other hand, the accumulation of senescent cells escaping immune clearance can promote tumor progression and drive tumor vascularization by the SASP, which recruits tumor-infiltrating MDSCs and senescent fibroblasts and promotes cancer stemness (Krtolica et al., 2001; Coppé et al., 2006; Jackson et al., 2012; Milanovic et al., 2018). Intriguingly, in the early stage of hepatic carcinoma, cellular senescence acts as a tumor suppressor, while in the late stage, SASP promotes tumor progression by inhibiting immune surveillance (Eggert et al., 2016). Therefore, it is necessary to further explore the role of cellular senescence in tumor immunity.

Long non-coding RNAs (lncRNAs) are composed of more than 200 nucleotides in length but do not encode proteins. LncRNAs play a vital role in the development of various tumors (Goodall and Wickramasinghe, 2021), and differentially expressed lncRNAs can affect the progression of gastric cancer and are potential markers of gastric cancer diagnosis, prognosis, and drug resistance (Yuan et al., 2020). Previous studies have shown that lncRNAs play an essential role in cellular senescence. For example, LINC00673 knockdown can trigger cellular senescence in a p53-dependent manner and inhibit lung cancer cell proliferation (Roth et al., 2018). Overexpression of lncRNA PLK4 inhibits tumor progression of hepatocellular carcinoma by promoting YAP-mediated cellular senescence (Jia et al., 2020). The NF-κB/HOTAIR (lncRNA) positive feedback loop contributes to cellular senescence in ovarian cancer (Özeş et al., 2016). The TCGA database contains transcriptome data and corresponding clinical data of 30 types of cancer (El-Arabey et al., 2020), and some researchers have constructed lncRNA signatures based on the TCGA database to predict the prognosis of tumor patients and guide individualized treatment (Qing et al., 2022). However, cellular senescence-related lncRNA in STAD has not yet been elucidated.

Our study established and validated a risk signature based on cellular senescence-related lncRNA and explored its prognostic value for STAD patients. Then, the differences in potential signaling pathways, TME, TMB, MSI, and TIDE scores between high- and low-risk groups were further analyzed. We expected our findings to provide a new perspective for predicting prognosis and individualized immunotherapy in STAD patients.

## Materials and methods

### Data collection

Gene expression profiles for 407 samples (32 normal stomach tissue samples and 375 stomach adenocarcinoma samples) and corresponding clinical and survival information were downloaded from the TCGA database (https://portal.gdc.cancer.gov/). After excluding samples with missing survival time and survival time less than 30 days, the entire set, including 337 STAD cases, was randomly divided into a training set (*n* = 169, Supplementary Table S1) and a testing set (*n* = 168, Supplementary Table S2). The training set was utilized to build the risk model, and the testing set and entire set were used to verify the risk model. A total of 279 cellular senescence-related genes were obtained from the CellAge database (https://genomics.senescence.info/cells/, Supplementary Table S3).

### Identification of differentially expressed cellular senescence-related lncRNAs (DECSlncRNAs) in STAD

Differentially expressed lncRNAs (DElncRNAs) and cellular senescence-related genes (DECSGs) between 32 normal stomach tissue samples and 375 stomach adenocarcinoma samples were obtained with adjusted *p* < 0.05 and | log2-fold change (FC)| > 1 using the R package limma (Ritchie et al., 2015). Next, Pearson’s correlation analysis was performed to screen cellular senescence-related lncRNAs (CSlncRNAs) based on DECSGs and lncRNAs with |*R*|>0.4 and *p* < 0.001. Finally, DECSlncRNAs were obtained by overlapping DElncRNAs and CSlncRNAs.

### Establishment and validation of the cellular senescence-related lncRNA prognostic signature for STAD

Univariate Cox regression analysis was performed to obtain potential prognostic DECSlncRNAs with the threshold of *p* < 0.05. Then, LASSO Cox regression analysis was executed to reduce overfitting lncRNAs with 10-fold cross-validation and 1,000 repeated times. The risk score of each STAD patient was calculated based on the expression levels and regression coefficients of cellular senescence-related lncRNAs. The formula was as follows: Risk score = *β*_lncRNA1_ × exp _lncRNA1_ + *ß*_lncRNA2_ × exp _lncRNA2_ + … + *ß*_lncRNAn_ × exp _lncRNAn_. Patients were divided into high- and low-risk groups based on the median risk score.

To compare overall survival (OS) between the high- and low-risk groups in training, testing , and the entire set, Kaplan–Meier survival analysis was performed using the R packages survminer and survival (Zhao et al., 2021). Subgroup analysis for the OS of STAD patients was also performed based on clinicopathological characteristics. ROC curves were constructed, and the area under the curve (AUC) values were calculated using the R package survival ROC (Heagerty et al., 2000).

### Independent prognostic and nomogram analysis

We performed univariate and multivariate Cox regression analyses to explore whether the risk score could be an independent prognostic factor for STAD patients. Age, gender, grade, clinical stage, tumor size (T), distance metastasis (M), lymph node metastasis (N), and risk score were included for analysis. To predict the survival of STAD patients at 1, 3, and 5 years, a nomogram integrating the risk score and clinicopathological factors was created using the R package rms (Xu et al., 2021). Calibration curves were plotted to detect the predictive performance of the nomograms for OS.

### Principal component analysis and gene set enrichment analysis

PCA is a commonly used unsupervised learning method that can reduce the dimension of multidimensional data and extract the main feature components of the data (Ringnér, 2008). To explore the distribution of high- and low-risk patients, we performed PCA based on the whole-genome, CSlncRNAs, and the CSLPS, including 15 cellular senescence-related lncRNAs.

GSEA software (version 4.1.0) was utilized to explore potential biological functions in high- and low-risk groups. The c2. cp.kegg.v7.4. symbols.gmt were used for annotated gene sets. A total of one thousand permutations were performed, and the normalized enrichment score (NES) was calculated based on the Affymetrix chip platform. Normal *p*-value < 0.05 and a false discovery rate (FDR *q*-value) < 0.25 were regarded as significantly enriched (Subramanian et al., 2005).

### Investigation of the immune landscape

The ESTIMATE algorithm was utilized to explore the difference in the TME between high- and low-risk groups. XCELL, TIMER, QUANTISEQ, MCPCOUNTER, EPIC, CIBERSORT-ABS, and CIBERSORT algorithms were utilized to analyze the correlation between immune components and risk scores based on CSLPS. Moreover, single-sample GSEA (ssGSEA) was used to analyze 16 infiltrating immune cells and 13 immune functions between high- and low-risk groups using the R package gsva (Hänzelmann et al., 2013). We also performed immune checkpoint-related gene differential expression analysis between the two subgroups.

### Immunotherapy response analysis

The mutation data of STAD patients were also downloaded from the TCGA database and analyzed using the R package Maftools (Mayakonda et al., 2018). The TMB difference between the high- and low-risk groups was compared. Then, patients were divided into high- and low-TMB groups according to the best cut-off TMB values. Survival analysis was performed based on tumor mutation burden status and risk score. Furthermore, we downloaded the microsatellite status data from TCIA (http://tcia.at/) and compared the differences between high- and low-risk groups. The tumor immune dysfunction and exclusion (TIDE) algorithm was utilized to explore the immunotherapy response in STAD patients using the website (http://tide.dfci.harvard.edu/).

### Statistical analysis

R software (version 4.1.2) was used for statistical analyses. The Wilcoxon test was used to compare clinicopathological characteristics, TME, TMB, MSI, and TIDE scores between high- and low-risk groups. The Kaplan–Meier curve was used to compare survival between different groups. Univariate and multivariate Cox regression analyses were used to analyze independent prognostic factors. ROC curves were used to assess the predictive power of the CSLPS. *p* < 0.05 was considered statistically significant. *, *p* < 0.05; **p < 0.01; ***p < 0.001.

## Results

### Identification of differentially expressed cellular senescence-related lncRNAs (DECSlncRNAs)

**FIGURE 1:**
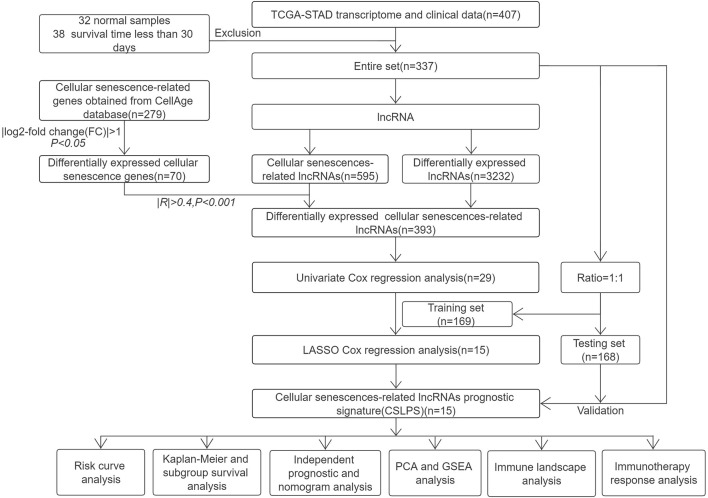
Flow chart of the study.

**FIGURE 2:**
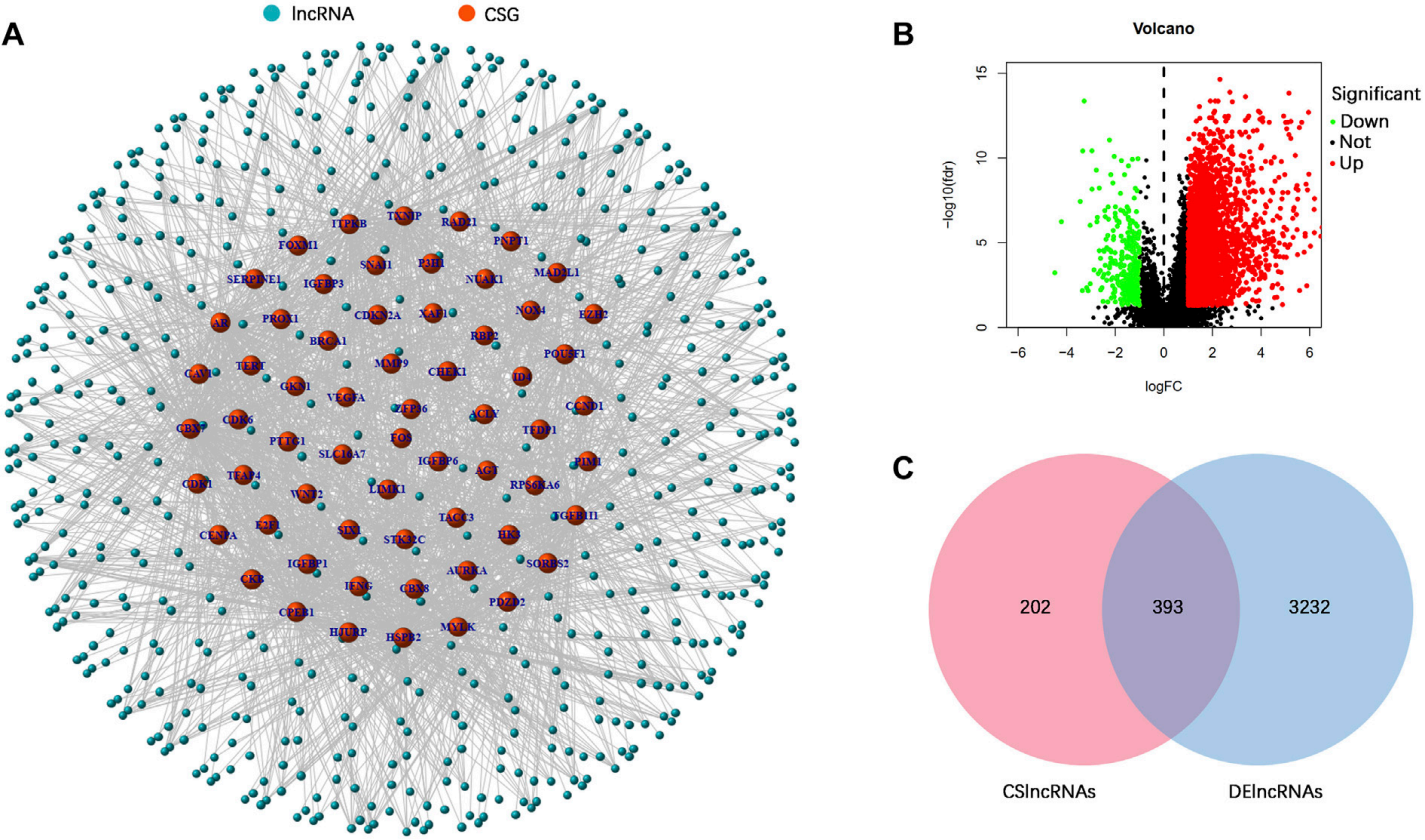
Identification of differentially expressed cellular senescence-related lncRNAs. **(A)** Coexpression analysis of lncRNAs and differentially expressed cellular senescence-related genes. **(B)** Volcano plot of differentially expressed lncRNAs in the TCGA–STAD dataset. **(C)** Venn diagram of DElncRNAs and cellular senescence-related lncRNAs.

The research process is shown in Figure 1. From the TCGA database, we downloaded 32 normal stomach tissue samples and 375 stomach adenocarcinoma samples. Then, the 279 genes obtained from the CellAge database were compared in 32 normal stomach tissue samples and 375 stomach adenocarcinoma samples to explore the expression of cellular senescence-related genes in STAD patients. Among them, 47 genes were upregulated, whereas 23 were downregulated (Supplementary Table S4). A total of 595 cellular senescence-related lncRNAs were obtained from coexpression analysis based on 70 cellular senescence-related genes *via* the criteria |*R*|>0.4 and *p* < 0.001 (Figure 2A; Supplementary Table S5). Then, 393 DECSlncRNAs were identified by overlapping with 3625 DElncRNAs in STAD (Figures 2B,C; Supplementary Table S6).

### Construction and validation of the cellular senescence-related lncRNA prognostic signature

**FIGURE 3:**
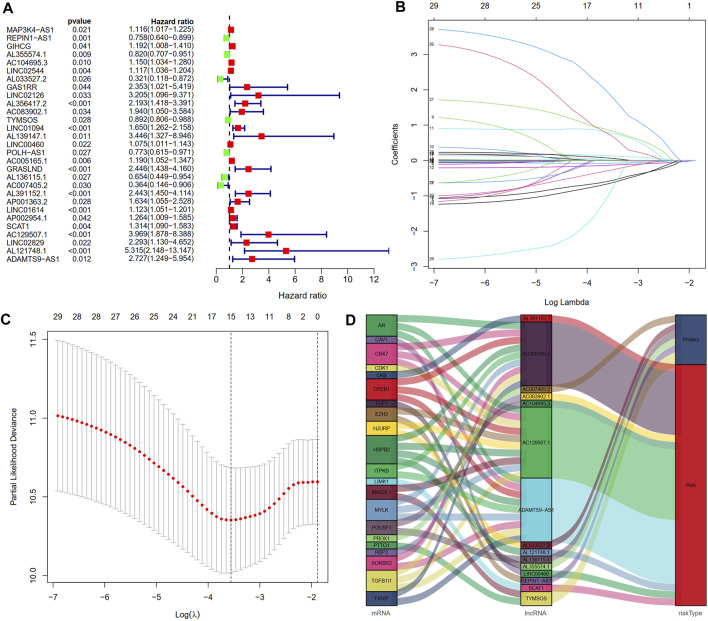
Construction of a cellular senescence-related lncRNA prognostic model. **(A)** Twenty-nine cellular senescence-related prognostic lncRNAs were obtained by univariate Cox regression analysis. **(B,C)** Cellular senescence-related lncRNA prognostic signature (CSLPS) was identified by the LASSO Cox regression analysis. **(D)** Sankey diagram of cellular senescence-related genes and lncRNAs.

After excluding samples with a survival time of less than 30 days, 337 out of 375 STAD samples were set as the entire set. No statistical differences in clinicopathological factors were observed between the training and testing sets (Table 1). Univariate Cox regression analysis showed that 29 lncRNAs were significantly associated with OS (*p* < 0.05, Figure 3A). To reduce overfitting of lncRNAs, LASSO Cox regression analysis was performed, and 15 of the 29 lncRNAs were chosen to construct CSLPS based on 1,000 times 10-fold cross-validation and the optimal value (Figures 3B,C; Table 2). The Sankey diagram displayed that six were protective and nine were risk lncRNAs (Figure 3D). Among them, AC007405.2, AL033527.2, AL136115.1, AL355574.1, REPIN1-AS1, and TYMSOS are potential protective factors, but AL391152.1, AC005165.1, AC083902.1, AC104695.3, AC129507.1, ADAMTS9-AS1, AL121748.1, LINC00460, and SCAT1 are underlying hazardous indicators. Risk score was calculated according to the formula: risk score = (−0.0056 × REPIN1-AS1) + (−0.0779 × AL355574.1) + (0.1341 × AC104695.3) + (−0.5803 × AL033527.2) + (0.7814 × AC083902.1) + (−0.1343 × TYMSOS) + (0.0129 × LINC00460) + (0.0104 × AC005165.1) + (−0.6483 × AL136115.1) + (−0.2790 × AC007405.2) + (0.8521 × AL391152.1) + (0.0659 × SCAT1) + (0.7297 × AC129507.1) + (1.8695 × AL121748.1) + (−0.9227 × ADAMTTS9-AS1).

**TABLE 1:** Clinical features in the training set, testing set, and entire set.

Variable	Type	Entire set (*n* = 337)	Training set (*n* = 169)	Testing set (*n* = 168)	*χ2*	*p* value
Age	>65	181	89	92	0.625	0.745
	≤65	153	79	74		
	unknown	3	1	2		
Gender	Female	119	61	58	0.091	0.763
	Male	218	108	110		
Grade	G1-2	129	70	59	1.694	0.432
	G3	199	94	105		
	unknown	9	5	4		
Stage	Stages I–II	152	75	77	0.315	0.854
	Stages III–IV	171	86	85		
	unknown	14	8	6		
T	T1-2	89	43	46	0.292	0.891
	T3-4	244	124	120		
	unknown	4	2	2		
M	M0	303	150	153	4.269	0.120
	M1	22	15	7		
	unknown	12	4	8		
N	N0	99	49	50	0.830	0.660
	N1-3	227	113	114		
	unknown	11	7	4		

**TABLE 2:** Regression coefficients of 15 cellular senescence-related lncRNAs.

LncRNA	Coef
REPIN1-AS1	−0.00558529468955258
AL355574.1	−0.0778920750784493
AC104695.3	0.134094653202654
AL033527.2	−0.580259682168754
AC083902.1	0.781408951971522
TYMSOS	−0.134336197045362
LINC00460	0.0129098342967563
AC005165.1	0.0103572444141003
AL136115.1	−0.648306232373688
AC007405.2	−0.279024723404953
AL391152.1	0.85206375703833
SCAT1	0.0659323135518391
AC129507.1	0.729691404665221
AL121748.1	1.86947552399675
ADAMTS9-AS1	−0.922683946724087

**FIGURE 4:**
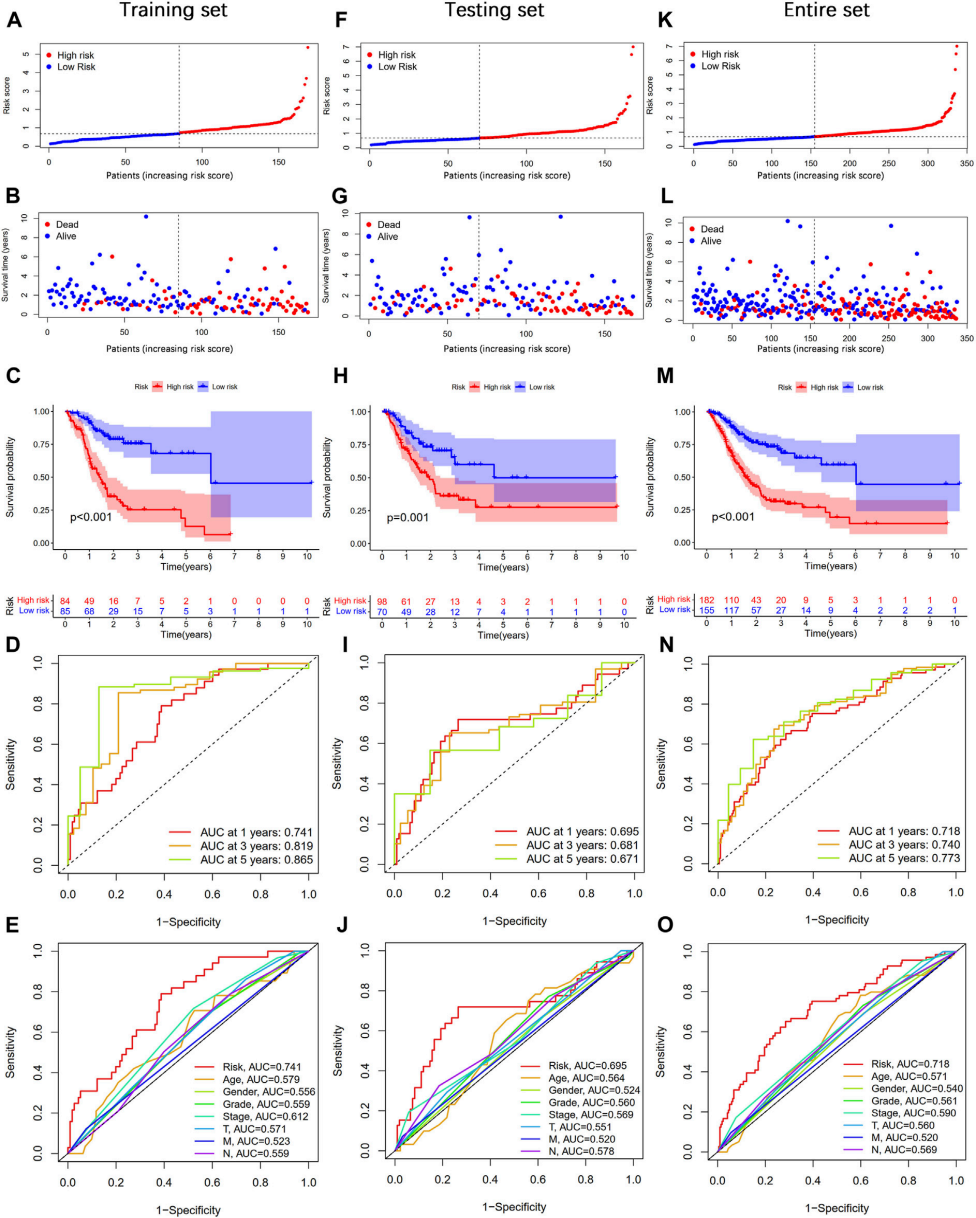
Prognosis value of the cellular senescence-related lncRNA prognostic signature (CSLPS). **(A)** Distribution of risk score, **(B)** survival status, **(C)** Kaplan–Meier survival curves, **(D)** the 1-, 2-, and 3-year ROC curves, **(E)** and the 1-year ROC curves of risk score and clinical characteristics in the training set. **(F–O)** Validation sets, including the testing set and the entire set, were analyzed similarly.

According to the median value of the risk score, STAD patients were divided into high- and low-risk groups. As shown in Figures 4A,B, the risk score was positively associated with the number of deaths. The Kaplan–Meier survival analysis indicated that STAD patients in the high-risk group had significantly shorter OS time than those in the low-risk group (Figure 4C). The 1-, 3-, and 5-year AUC values were 0.741, 0.819, and 0.865, respectively (Figure 4D). Moreover, the AUC value of the risk score at 1 year was higher than that of age, gender, grade, stage, T, M, and N (Figure 4E). At the same time, we performed the same analysis in two validation sets. Similar results were observed in the testing and entire sets (Figures 4F–O). Taken together, our established CSLPS shows good performance in predicting survival outcomes of STAD patients.

### Subgroup survival analysis

**FIGURE 5:**
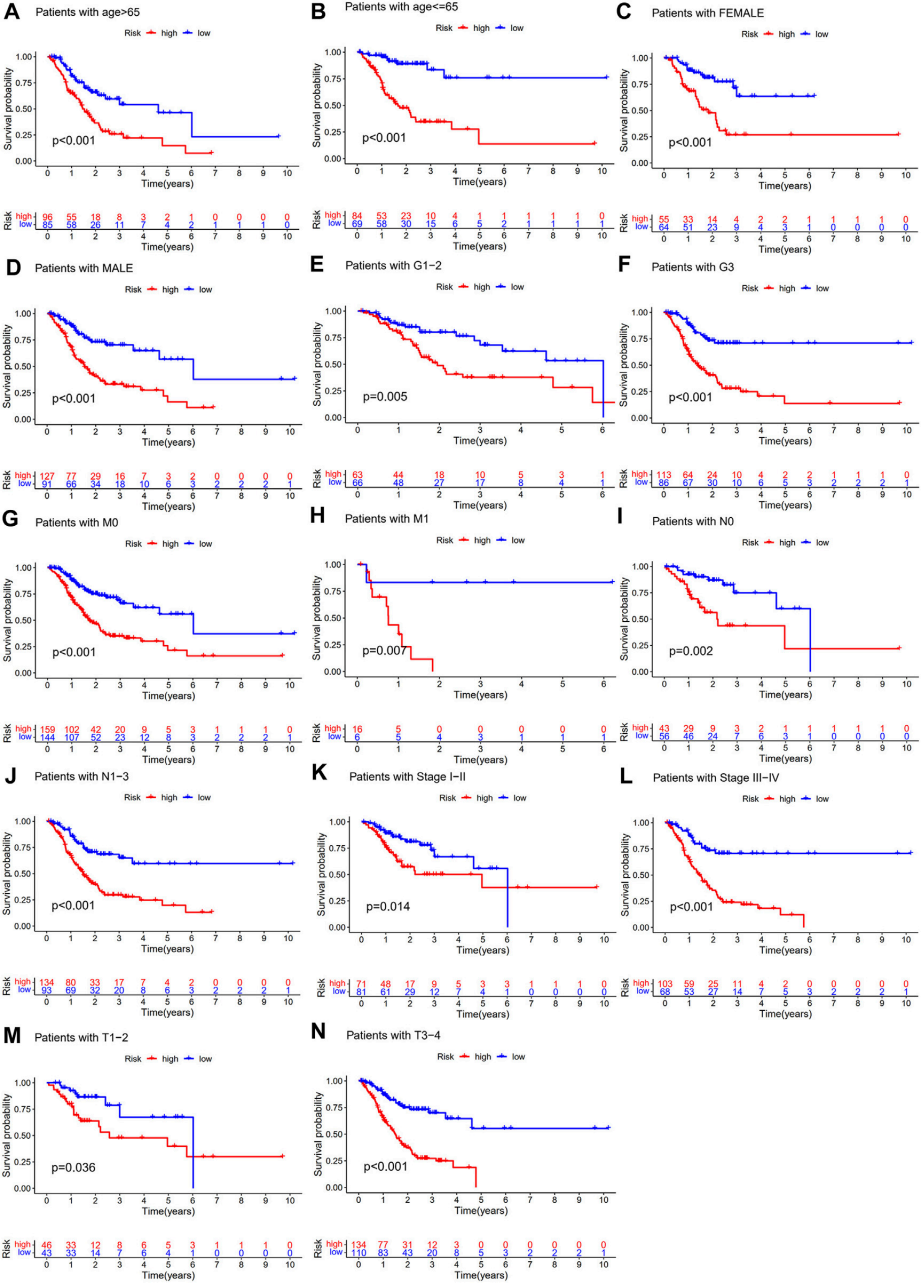
Subgroup survival analysis of the cellular senescence-related lncRNA prognostic model. Kaplan–Meier survival analysis for **(A)** age >65 years, **(B)** age ≤ 65 years, **(C)** female, **(D)** male, **(E)** G1-2, **(F)** G3, **(G)** M0, **(H)** M1, **(I)** N0, **(J)** N1-3, **(K)** stages I–II, **(L)** stages III–IV, **(M)** T1-2, and **(N)** T3-4 between high- and low-risk groups.

To further explore whether the CSLPS was associated with the clinicopathological features of STAD patients, we performed a subgroup survival analysis. The subgroups were divided by age (>65 years or ≤ 65 years), sex (female or male), grade (G1-2 or G3), M stage (M0 or M1), N stage (N0 or N1-3), TNM stage (stages I–II or stages III–IV), and T stage (T1-2 or T3-4). We found that the OS time of high-risk group STAD patients was significantly shorter than that of low-risk group STAD patients in all the subgroups (Figure 5).

### Independent prognostic and nomogram analysis

**FIGURE 6:**
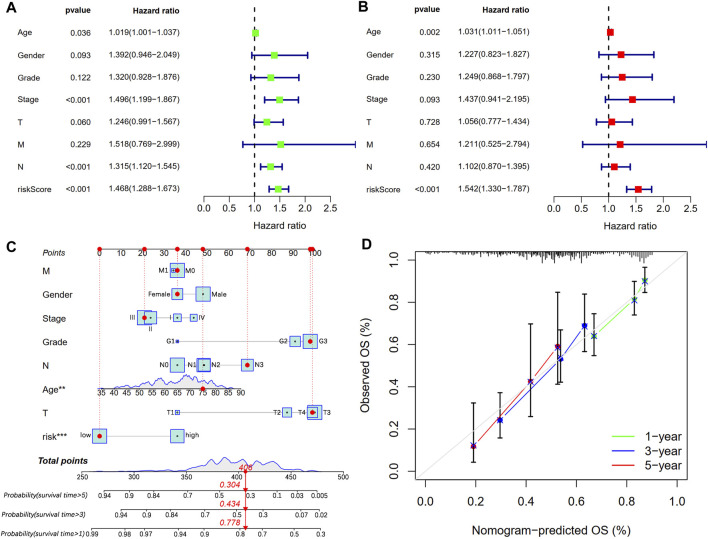
Independent prognostic analysis and prognostic nomogram establishment. **(A)** Univariate and **(B)** multivariate Cox regression analyses of clinical features and risk score with OS. **(C)** Nomogram to predict STAD patients’ outcomes in 1, 3, and 5 years. **(D)** Calibration curves for 1-, 3-, and 5-year OS.

We conducted univariate and multivariate Cox regression analyses to explore whether CSLPS could be an independent prognostic factor for STAD patients. Univariate Cox regression analysis showed that age (HR = 1.019, 1.001–1.037, *p* = 0.036), stage (HR = 1.496, 1.199–1.867, *p* < 0.001), N stage (HR = 1.315, 1.120–1.545, *p* < 0.001), and risk score (HR = 1.468, 1.288–1.673, *p* < 0.001) predicted worse OS (Figure 6A). Furthermore, multivariate Cox regression analysis verified that the risk score (HR = 1.542, 1.330–1.787, *p* < 0.001) based on CSLPS was an independent prognostic factor in STAD patients (Figure 6B). To further improve the predictive value of the CSLPS in STAD patients, we constructed a nomogram taking into account age, gender, stage, grade, T, N, M, and risk score to predict OS at 1, 3, and 5 years (Figure 6C). The 1-, 3-, and 5-year calibration curves demonstrated good agreement between predicted and observed OS (Figure 6D).

### Principal component analysis and gene set enrichment analysis

**FIGURE 7:**
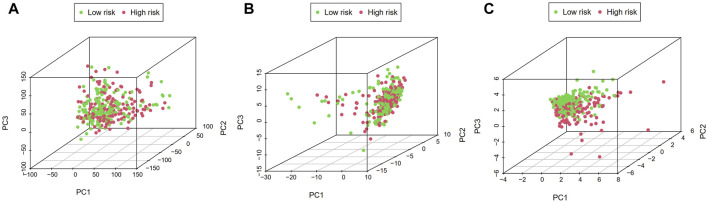
Principal component analysis (PCA). Distribution of high- and low-risk groups based on the **(A)** whole-genome, **(B)** cellular senescence-related lncRNAs, and **(C)** the risk model including fifteen cellular senescence-related lncRNAs.

**FIGURE 8:**
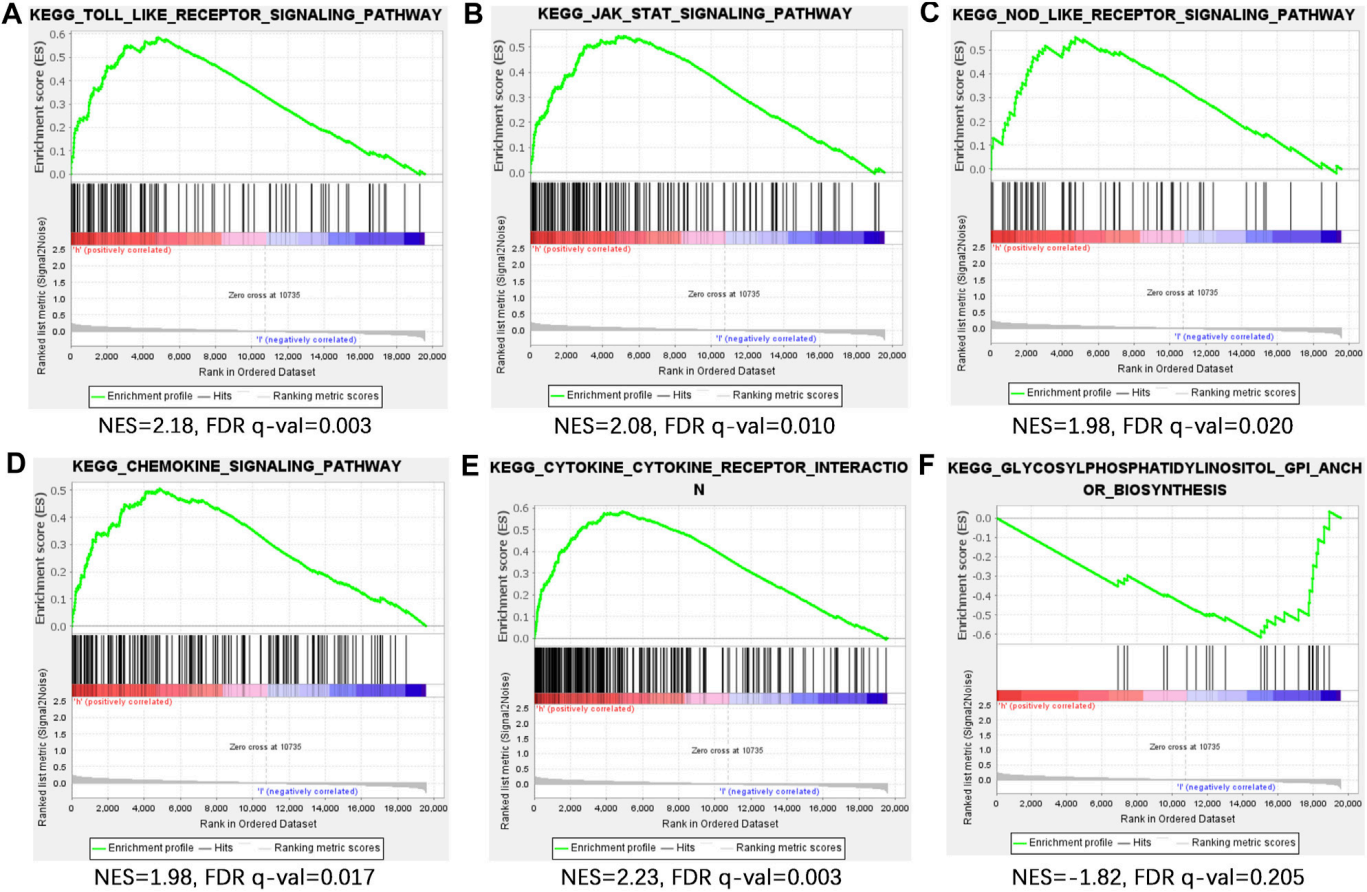
Gene set enrichment analysis (GSEA) of CSLPS. **(A)** Toll-like receptor signaling pathway, **(B)** JAK/STAT signaling pathway, **(C)** NOD-like receptor signaling pathway, **(D)** chemokine signaling pathway, **(E)** and cytokine–cytokine receptor interaction were activated in the high-risk group. **(F)** Glycosylphosphatidylinositol GPI anchor biosynthesis was activated in the low-risk group.

PCA visualization analysis based on the whole genome and CSlncRNAs showed that the distribution of the high-risk group and the low-risk group was scattered (Figures 7A,B), while visualization analysis based on the 15 lncRNAs in CSLPS showed that the high- and low-risk groups had significantly different distributions (Figure 7C). PCA further verified the grouping ability of CSLPS, including 15 CSlncRNAs. Next, GSEA was utilized to explore the potential biological functions of patients in high- and low-risk groups based on the CSLPS. The results suggested that the high-risk group was associated with the Toll-like receptor signaling pathway, JAK/STAT signaling pathway, NOD-like receptor signaling pathway, chemokine signaling pathway, and cytokine–cytokine receptor interaction (Figures 8A–E). In contrast, the low-risk group was related to glycosylphosphatidylinositol GPI anchor biosynthesis (Figure 8F).

### Immune landscape analysis

**FIGURE 9:**
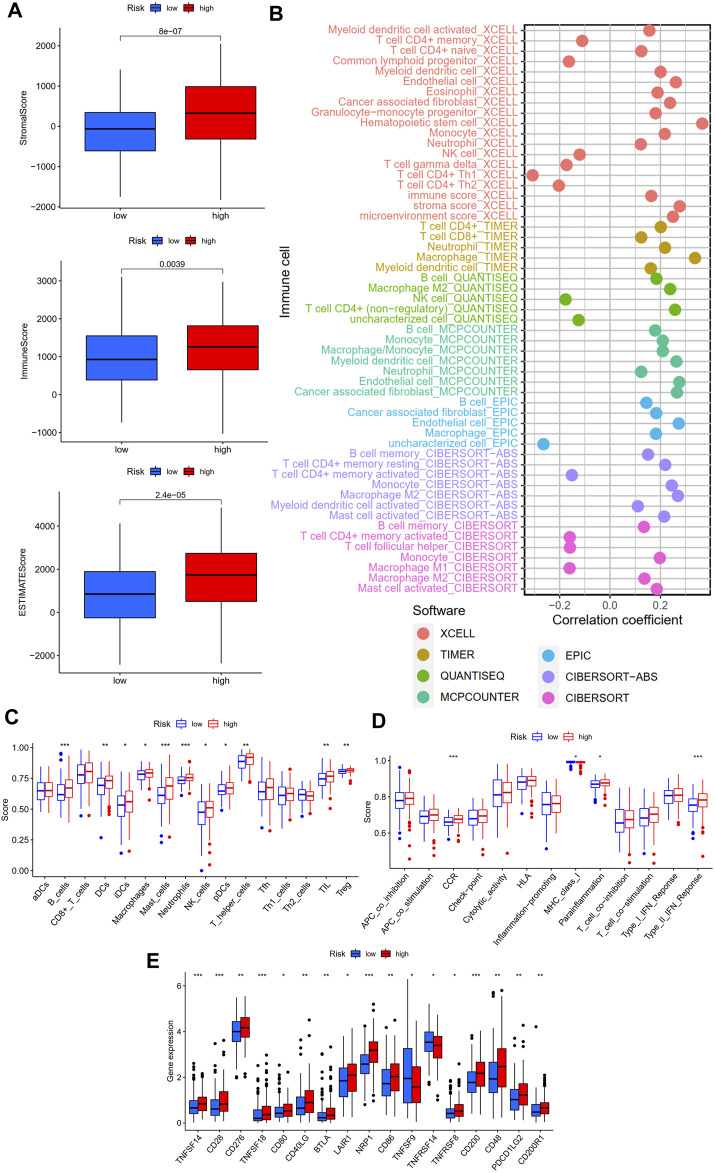
Immune landscape of the cellular senescence-related lncRNAs model. **(A)** Tumor microenvironment analysis between high- and low-risk groups by ESTIMATE. **(B)** Correlation analysis of immune components and risk scores based on XCELL, TIMER, QUANTISEQ, MCPCOUNTER, EPIC, CIBERSORT-ABS, and CIBERSORT algorithms. **(C)** Sixteen immune cells and **(D)** 13 immune-related functions between the high- and low-risk groups by ssGSEA. **(E)** Expression of immune checkpoint-related genes between the high- and low-risk groups. *p < 0.05; **, *p* < 0.01; ***p < 0.001.

To explore the relevance of our established CSLPS to the immune landscape, we first explored differences in the TME between high- and low-risk groups. ESTIMATE analysis showed that the high-risk group had higher stromal, immune, and ESTIMATE scores (Figure 9A). Then, we explored the correlation between risk scores and immune cell infiltration. A bubble chart based on seven different algorithms showed that the risk score was positively correlated with myeloid dendritic cells, cancer-associated fibroblasts, M2 macrophages, B cells, hematopoietic stem cells, T cell CD8^+^, and mast cells while negatively correlated with NK cells, M1 macrophage, T cell CD4^+^ Th1, and T cell CD4^+^ Th2 (all *p* < 0.05, Figure 9B, Supplementary Table S7). In addition, ssGESA was applied to explore the difference between the two subgroups of 16 immune cells and 13 immune-related pathways. We found that B cells, DCs, iDCs, macrophages, mast cells, neutrophils, NK cells, pDCs, T helper cells, TIL, Treg, CCR, parainflammation, and type Ⅱ IFN response were more enriched in the high-risk group, while the MHC class Ⅰ is less enriched in the high-risk group (Figures 9C,D). Finally, we analyzed the expression levels of the immune checkpoint-related genes between the two subgroups. The results showed that TNFSF14, CD28, CD276, TNFSF18, CD80, CD40LG, BTLA, LAIR1, NRP1, CD86, TNFRSF8, CD200, CD48, PDCD1LG2, and CD200R1 genes were more highly expressed in the high-risk group, while TNFSF9 and TNFRSF14 were lower expressed in the high-risk group (Figure 9E). The aforementioned findings indicate that high-risk group patients present an immunosuppressive microenvironment.

### Immunotherapy response analysis

**FIGURE 10:**
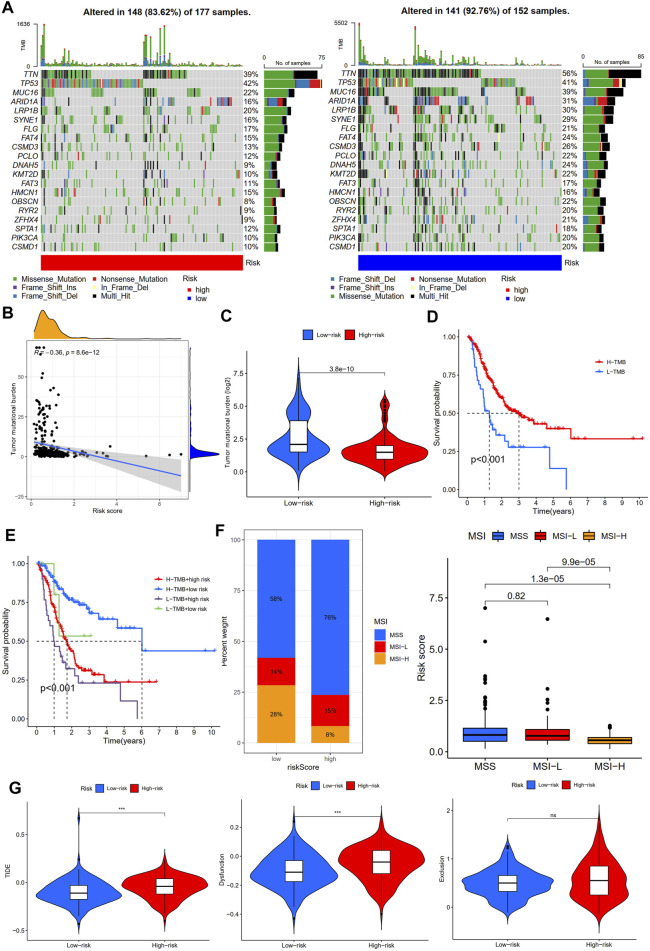
Immunotherapy response analysis between high- and low-risk groups. **(A)** Mutation profile of STAD patients in high- and low-risk groups. **(B)** Correlation analysis of TMB and risk scores. **(C)** Tumor mutation burden analysis between high- and low-risk groups. **(D)** Survival analysis between high- and low-tumor mutation burden (TMB) in STAD patients. **(E)** Survival analysis based on tumor mutation burden status and the risk score. **(F)** Microsatellite instability (MSI) analysis of STAD patients in high- and low-risk groups. **(G)** TIDE, dysfunction, and exclusion scores between high- and low-risk groups.

TMB and MSI were considered predictive biomarkers of tumor immunotherapy response (Cristescu et al., 2018; Liu et al., 2019). As shown in Figure 10A, the most common type of mutation in high- and low-risk group patients was missense mutation, and the top three mutated genes were TTN, TP53, and MUC16. Intriguingly, TTN and MUC16 were more likely to be mutated in the low-risk group than in the high-risk group. Moreover, TMB was negatively associated with risk scores, and STAD patients in high-risk groups had a lower TMB than those in low-risk groups (Figures 10B,C). Survival analysis showed that STAD patients with a high TMB had better outcomes than those with a low TMB (Figure 10D), and the risk score reduced the prognostic value in the high-TMB group according to the survival analysis combined TMB and risk score (Figure 10E). In addition, the low-risk group had a lower proportion of patients with microsatellite stability (MSS) and a higher proportion of patients with high microsatellite instability (MSI-H) (Figure 10F; Supplementary Table S8). In addition, the TIDE score was a novel valuable predictive biomarker for tumor immunotherapy response, and patients with lower TIDE scores could benefit from immunotherapy and have a longer survival time (Jiang et al., 2018). Interestingly, we found that STAD patients in the low-risk group had lower TIDE scores and T-cell dysfunction scores than those in the high-risk group, but no statistical difference between the two subgroups in T cell exclusion was found (Figure 10G). The aforementioned results suggest that the low-risk group of STAD patients may be more effective for immunotherapy.

## Discussion

STAD is a common malignant tumor of the digestive system with insidious onset and high mortality (Sung et al., 2021). Cellular senescence, a new hallmark of cancer, displays both antitumor and pro-tumor activity (Hanahan, 2022). LncRNA-related risk signatures have recently become a research hotspot due to their excellent predictive performance (Wang et al., 2021; Qing et al., 2022). However, prognostic signature based on cellular senescence-related lncRNAs has remained unexplored in STAD.

Our study first identified 393 differentially expressed cellular senescence-related lncRNAs through differential expression analysis and correlation analysis. Then, a prognostic signature containing fifteen lncRNAs was established using univariate Cox regression analysis and LASSO Cox regression analysis. Survival analysis and ROC curve analysis demonstrated that the prognostic signature has an excellent prognostic predictive ability in STAD in the training set. Meanwhile, we performed the same analysis in the two validation sets, namely, the testing set and the entire set and surprisingly obtained similar results. In addition, our subgroup survival analysis showed shorter overall survival in high-risk groups in each clinical subgroup. Furthermore, univariate and multivariate regression analyses showed that the risk score based on the fifteen lncRNAs was an independent prognostic factor for STAD patients. Immediately afterward, we constructed a nomogram using the risk score and clinicopathological characteristics, which can accurately and reliably predict the 1-, 3-, and 5-year survival rates of STAD patients. In recent years, ferroptosis and immune-related lncRNA prognostic models have shown good predictive ability (Guo et al., 2021; Yuan et al., 2021), and the prognostic model based on cellular senescence-related lncRNAs in this study has the same excellent predictive performance, which not only demonstrates the reliability of our study but also provides a basis for further exploring the role of the lncRNA/mRNA regulatory network in tumor cellular senescence.

Our established novel prognostic signature consists of fifteen cellular senescence-related lncRNAs. AC007405.2, AL033527.2, and AC083902.1 were identified for the first time. AL136115.1, AL355574.1, and REPIN1-AS1 were reported as favorable underlying factors for STAD patients (Chen et al., 2021; Zhang et al., 2021; Luo et al., 2022), while AL391152.1 (Liu et al., 2020), AC005165.1 (Wang et al., 2022), AC104695.3 (Zhang et al., 2021), and AC129507.1 (Zha et al., 2021) were risk lncRNAs for STAD patients, which is consistent with our results. ADAMTS9-AS1 can inhibit apoptosis and autophagy through the PI3K/AKT/mTOR signaling pathway and promote bladder cancer progression (Yang et al., 2021) while inhibiting colon cancer cell progression through the Wnt/β-catenin signaling pathway (Li et al., 2020). Curiously, ADAMTS9-AS1 has also promoted colorectal cancer cell proliferation and epithelial–mesenchymal transition (EMT) (Chen et al., 2020). However, there is no relevant research on ADAMTS9-AS1 in STAD. Lai et al. (2021) demonstrated that AL121748.1 was a ferroptosis-related lncRNA associated with immunotherapy responses in STAD patients. LINC00460 may play oncogenic roles and serve as a potential prognostic biomarker in various tumors (Dai et al., 2020), including colorectal cancer, gastric cancer, head and neck squamous cell carcinoma, and hepatocellular carcinoma (Jiang et al., 2019; Tu et al., 2019; Yang et al., 2020; Hou et al., 2021). In colorectal cancer, LINC00460 was overexpressed and promoted proliferation, migration, and invasion by enhancing HMGA1 mRNA stability (Hou et al., 2021). In gastric cancer, LINC00460 promoted tumor progression by silencing CCNG2 in an EZH2/LSD1-dependent manner (Yang et al., 2020). AL139352.1 and AL121748.1 were reported as risk factors and associated with poor prognosis in gastric cancer (Liu et al., 2020; Lai et al., 2021), which is consistent with our findings. Zhang et al. (2020) reported that a three-lncRNA signature, including SCAT1, could predict pathological response and outcome for esophageal squamous cell carcinoma patients.

To further explore the potential biological functions of the risk model, we first performed a PCA analysis. The results showed that STAD patients could be more clearly divided into two subgroups based on fifteen cellular senescence-related lncRNAs, demonstrating the prognostic signature’s superiority. Next, GSEA was performed to investigate the difference between the two subgroups. We found that the Toll-like receptor signaling pathway, JAK/STAT signaling pathway, NOD-like receptor signaling pathway, chemokine signaling pathway, and cytokine–cytokine receptor interaction were enriched in high-risk groups. The Toll-like receptor signaling pathway played a critical role in the innate and adaptive immune system and was expected to be a novel strategy for tumor immunotherapy (Aluri et al., 2021; Nouri et al., 2021). Interestingly, Hari et al. found that Toll-like receptor 2 could control the senescence-associated secretory phenotype (Hari et al., 2019). Similarly, Toll-like receptor 4 knockdown decreased cellular senescence by S100A9 (Shi et al., 2019). Other signaling pathways enriched in the high-risk groups were also related to immune regulation (Caruso et al., 2014; Griffith et al., 2014; Yoshimura et al., 2018). Therefore, we further explored the immune infiltration between high- and low-risk groups. We found that the high-risk group had higher stromal, immune, and ESTIMATE scores, indicating a different tumor microenvironment from the low-risk group. Macrophages are divided into two types, M1 and M2. Studies have shown that M1 macrophages can promote inflammatory responses and exert antitumor effects, while M2 macrophages induce an immunosuppressive microenvironment and promote tumor progression (Pan et al., 2020). Our findings showed that risk scores were positively correlated with M2 macrophages and negatively correlated with M1 macrophages. Patients with high-risk scores had shorter overall survival, which may be related to M2 macrophage infiltration. Furthermore, risk scores were positively correlated with cancer-associated fibroblasts (CAFs). CAFs interact with tumor-infiltrating immune cells by secreting various cytokines, growth factors, chemokines, exosomes, and other effector molecules, thereby forming an immunosuppressive TME, enabling cancer cells to evade the immune surveillance system (Liao et al., 2019). Studies have shown that MHC class I is a second “don’t eat me” signal on the surface of cancer cells, and low expression of MHC class I antigens hinders antigen presentation and promotes tumor cell immune escape (Yamamoto et al., 2020). Our study found that STAD patients in the high-risk group had lower expression of MHC class I antigens, suggesting that the poor clinical outcomes of STAD patients in the high-risk group might be related to the immune escape of tumor cells.

In recent years, targeted immune checkpoint therapy has been a milestone in treating gastric cancer, but the response rate of the overall population to immunotherapy is not high (Zhao et al., 2019). Therefore, screening out the population with a high response to immunotherapy is necessary for more precise treatment. Studies have shown that TMB, MSI, and TIDE scores are predictive markers of immunotherapy efficacy, and patients with high TMB, MSI-H, and low TIDE scores have a better response to immunotherapy and have more prolonged survival (Cristescu et al., 2018; Jiang et al., 2018; Liu et al., 2019). Studies have shown that when there is a loss of mismatch repair gene function in tumor cells, the wrongly replicated DNA cannot be repaired in time, which increases TMB and generates neoantigens (Picard et al., 2020). Immune cells can effectively recognize tumor cell neoantigens and form tumor-infiltrating lymphocytes, thereby inhibiting tumor growth. Our study found that the risk score was inversely correlated with the TMB, and patients in the low-risk group had a higher TMB. Therefore, we speculate that the more prolonged survival of STAD patients with a high TMB may be related to the antitumor immunity caused by more neoantigens. Survival analysis showed that the risk score based on CSLPS could affect the survival of STAD patients independently of TMB, which further demonstrated that the risk score was an independent prognostic factor for STAD patients and was consistent with the previous multivariate regression analysis. In addition, patients in the low-risk group had higher proportions of MSI-H and lower TIDE scores. The aforementioned results demonstrated that the risk score could reflect the immunotherapy response to a certain extent. Thus, our prognostic signature based on cellular senescence-related lncRNAs may provide novel perspectives in screening high-benefit populations of immunotherapy.

Our study has the following advantages: First, we screened out the differentially expressed and prognosis-related cellular senescence-related lncRNAs in STAD, which will provide clues for the subsequent exploration of the mechanism of lncRNAs in senescence. Second, we found significant differences in the tumor immune microenvironment between high- and low-risk groups, and patients in the high-risk group presented an immunosuppressive microenvironment, which provided a direction for exploring the reasons for the short overall survival of high-risk patients. Third, immunotherapy response analysis found that patients in the low-risk group had a higher response rate to immune checkpoint therapy, which would provide a reference for the selection of immunotherapy for STAD patients.

There are several limitations to our study: First, external verification and additional clinical STAD patients are needed further to confirm the performance of our established prognostic signature. Moreover, *in vivo* and *in vitro* experiments are required to understand the relationship between risk scores and TME, TMB, and MSI.

## Conclusion

In conclusion, we developed a novel cellular senescence-related lncRNA prognostic signature, which could accurately predict the prognosis for STAD patients. Furthermore, low-risk groups displayed higher TMB, a higher proportion of MSI-H, and lower TIDE scores, implying more sensitivity to immunotherapy.

## Data Availability

Publicly available datasets were analyzed in this study. The names of the repository/repositories and accession number(s) can be found in the article/Supplementary Material.
